# Association between Obesity and Puberty Timing: A Systematic Review and Meta-Analysis

**DOI:** 10.3390/ijerph14101266

**Published:** 2017-10-24

**Authors:** Wenyan Li, Qin Liu, Xu Deng, Yiwen Chen, Shudan Liu, Mary Story

**Affiliations:** 1 School of Public Health and Management, Research Center for Medicine and Social Development, Collaborative Innovation Center of Social Risks Governance in Health, Chongqing Medical University, Chongqing 400016, China; lwy562011@163.com (W.L.); dengxuroy@163.com (X.D.); m13983816170@163.com (Y.C.); liushudankelly@163.com (S.L.); 2 Department of Community and Family Medicine, Duke University, Durham, NC 27708, USA; mary.story@duke.edu; 3 Duke Global Health Institute, Duke University, Durham, NC 27708, USA

**Keywords:** childhood obesity, puberty timing, systematic reviews, meta-analysis

## Abstract

This systematic review and meta-analysis examined the associations between obesity and puberty timing based on scientific evidence. Eight electronic databases were searched up to February 2017 for eligible studies, and two reviewers screened the articles and extracted the data independently. A total of 11 cohort studies with 4841 subjects met the inclusion criteria. Compared with the group of normal-weight girls, the obese group had more girls with menarche (RR: 1.87, 95% CI: 1.59–2.19, 2 studies). The number of girls with early puberty was significantly higher in the obese group than the normal weight group (RR: 2.44, 95% CI: 1.32–4.52, 5 studies). However, no differences were detected between girls who were obese or normal weight at age of menarche (WMD: −0.53 years, 95% CI: −1.24–0.19, 2 studies). There is no consistent result in the relationship between obesity and timing of pubertal onset in boys. Obesity may contribute to early onset of puberty in girls, while in boys, there is insufficient data. Given the limited number of cohort studies included in this meta-analysis, high-quality studies with strong markers of puberty onset, as well as standardized criteria for defining obesity are needed.

## 1. Introduction

Puberty is initiated in late childhood through a cascade of endocrine changes that lead to sexual maturation and reproductive capability [1]. The onset of puberty is reflected by the appearance of breast buds (B2) in girls [2] and genital changes (G2) in boys [3] as described by Tanner definitions [4]. Puberty timing is a relative concept, referring to an individual’s status as a referent group or a set of norms [5]. There has been a secular trend of early puberty timing since the late 19th century, reflected by a lower mean age of menarche in most developed countries, and in recent years, also in developing countries [6]. Previous studies showed that early puberty may lead to a number of adverse outcomes such as adolescent risk-taking behaviors, short adult stature, higher adult body mass index (BMI), waist circumference and adiposity, increased risk of adult-onset diabetes and pre-menopausal breast cancer [7,8].

Race, nutrition, genetic and environmental factors all impact puberty timing [9]. Multiple studies have shown that the secular trend of puberty timing in girls is related with the increasing trend of childhood obesity. Longitudinal studies have found that girls with higher body fat reveal an earlier pubertal development [10,11,12,13,14]. Several cross-sectional studies have shown a significant correlation between feminine obesity and earlier menarcheal age [15,16,17,18], pubertal growth spurt, earlier development of peak height velocity (PHV) [19] and breast and pubic hair [20]. However, other studies have failed to find any association [21,22]. Furthermore, the findings among boys have been inconsistent. One US study with a large and racially diverse sample of boys [23] found evidence of earlier puberty for overweight compared with normal or obese boys, and later puberty for boys who were obese compared with normal and overweight boys. The data reported by Wang [24] with a sample of 1520 US boys showed that compared with their counterparts, early maturing boys were thinner.

In view of the fact that the relationship between obesity and puberty timing has been inconsistent, we conducted a systematic review and meta-analysis to identify the associations between obesity and puberty timing in both girls and boys. To our knowledge, this is the first systematic review and meta-analysis on child obesity and the timing of puberty in girls and boys. 

## 2. Methods

### 2.1. Search Strategy

We searched the following databases up to February 2017: Cochrane Library, PubMed, ISI, OVID, EBSCO, CNKI, VIP and WANGFANG DATA. Original studies were searched using both the MeSH terms and free terms “obesity” or “obese” or “adiposity” or “overweight” or “bodyweight” or “BMI” or “body mass index” or “body fat” or “body fat mass”, in combination with “pubertal timing” or “puberty timing” or “sexual precocity” or “sexual prematurity” or “precocious puberty” or “premature pubarche” or “premature thelarche” or “menarche” or “first spermatorrhea”. All the retrieved publications were entered into reference-manager software (EndNote X7, Thomson Scientific, Stamford, CT, USA).

### 2.2. Selection Criteria

Studies were considered eligible for this systematic review and meta-analysis if they met the following inclusion criteria: (i) cohort studies; (ii) participants: children and adolescents; (iii) exposure group were obese children as defined by authors, and the control group were children with normal weight; (iv) main outcome measures were occurrence of secondary sexual characteristics, including age at pubertal events (menarche for girls and first spermatorrhea for boys), the number of cases (early development of breast, pubic hair and armpit hair) occurring in adolescence and the number of cases of precocious puberty. We excluded studies if: (i) children had any diseases which would affect pubertal development, such as congenital gonadal dysplasia, iodine deficiency disorders; (ii) The studies did not report pubertal development data for obesity and control groups but only analyzed the correlation between BMI (Body Mass Index) values and pubertal development; and (iii) studies defined the pubertal development by childhood growth (e.g., height growth spurt) rather than secondary sexual characteristics.

### 2.3. Data Screening and Extraction 

Two reviewers screened the retrieved literatures and citations by title and abstracts and then full texts independently based on inclusion criteria. Data was crosschecked and disagreements were solved by discussion or by revolving a third author. Then coding sheets in Microsoft Excel 2013 were developed before data extraction. The following information were extracted from each included study: general information, including author, country, year of publication, study design, sample size, participants’ characteristics (age and gender), the definition criteria of obesity and early puberty timing, as well as outcome measures. This information was extracted by two reviewers independently and disagreements were resolved by consensus or consultation with a third author.

### 2.4. Risk of Bias Assessment

The risk of bias of included studies was assessed based on the Newcastle-Ottawa Scale (NOS) [25] for cohort studies by two reviewers, independently. The third reviewer was involved when any disagreements existed. A score of 0–9 was used to evaluate included studies on the following items: selection of the study groups, between-group comparability and ascertainment the outcome for cohort studies. Based on the NOS, a study can be awarded a maximum of one score for items within the selection and outcome categories and a maximum of two scores for comparability. Studies are then divided into three grades: Grade A (scored 7–9, high quality), Grade B (scored 4–6, medium quality), Grade C (scored 0–3, low quality) [26]. The quality scores did not affect studies for inclusion, but was considered when performing sensitivity analysis and interpreting our research results.

### 2.5. Statistical Analysis

Data was combined using Review Manager Software (Version 5.3, Cochrane Collaboration, London, UK). Continuous and dichotomous outcomes were assessed as weighted mean difference (WMD) and risk ratio (RR) with 95% confidence intervals (CIs), respectively. Heterogeneity among studies was assessed statistically using the standard chi-square tests and I^2^ values. Once the effect was found to be heterogeneous (I^2^ > 20% or *p* < 0.05), the random effects model was used. Otherwise, the fixed effects model was used. Subgroup analysis was conducted by different outcome measures and definitions of early puberty. Sensitivity analyses were conducted to investigate the robustness of the results with the leave-one-out method. Statistical assessment of potential publication bias was not possible given the limited number of eligible trials [27].

## 3. Results

### 3.1. Search Results

**Figure 1:**
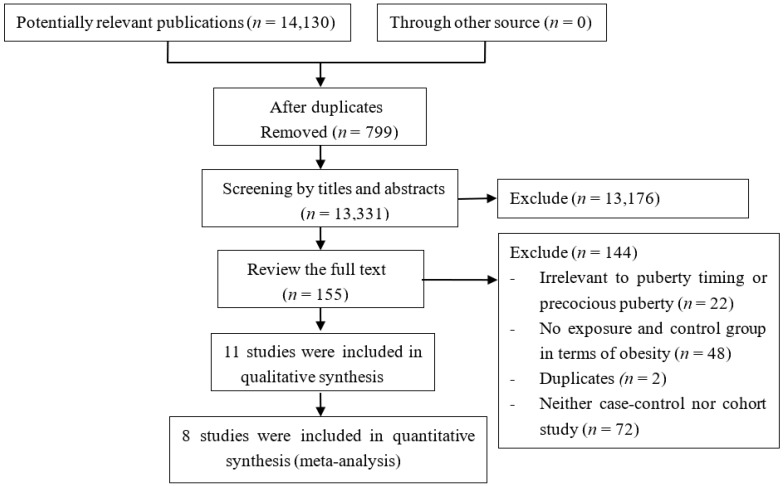
Flow diagram of the literature search.

The present search strategy identified 14,130 records. After duplicate checking, 13,176 out of 13,331 studies were excluded after the initial screening of titles and abstracts. Finally, eleven cohort studies [11,12,13,28,29,30,31,32,33,34,35] involving 4841 participants were included in the present study. The other 144 studies were excluded because they were not relevant to puberty timing, had no exposure group and control group in terms of obesity, were a duplicate publication, or were neither a case-control nor cohort study. A flow diagram of the selection process for the inclusion of studies is shown in Figure 1. 

### 3.2. Characteristics of Included Studies

Primary characteristics of included studies are summarized in Table 1 and Table 2. The included studies were published between 2003 and 2017, with sample sizes ranging from 80 to 1025. Of the 11 studies, four studies were conducted in US [11,12,13,34], two in China [28,30] and one study each in Spain [29], Iran [31], Portugal [32], Canada [33], Australia [35]. Of the eleven studies, ten were only girls and one was only with boys. The included studies in meta-analysis used five different criteria for defining obesity, including: (1) ≥95th percentile of BMI [11,28,31,33]; (2) criteria according to Cole [29]; (3) percentile of body fat by using skin-fold thickness ≥25% [30]; (4) ≥30th percentile of body fat [32]; (5)according to 2000 growth charts from the Centers for Disease Control and Prevention [12] The controls were mainly healthy populations that were matched with the obese group in age, gender, ethnicity and living environments. Of the 11 studies, five reported the number of youth with early puberty [11,12,30,32,33], four reported the outcome of age at menarche [13,29,31,35], two assessed the number of girls that had menarche [28,30], one reported number of girls with breast development stage 2 [30] and another one reported the genitalia development in boys [34].

### 3.3. Risk of Bias in Included Studies 

Among the eleven cohort studies, all were assessed as low risk of bias (scores of 7–9) (Table 3). All studies had adequate representativeness of the expose cohort (the obese group), selection of the non-exposed cohort (normal weight group) and ascertainment of exposure. All outcomes of interest were not presented at the start of the study except two studies [30,33]. For comparability, subjects in the obese and normal weight control groups were matched by age in eleven studies, while some studies also matched by height and socioeconomic status. All studies were followed long enough for the main outcomes to occur and had adequate time for follow up except for three studies [12,34,35].

### 3.4. Data Synthesis

#### Age at Menarche

**Figure 2:**
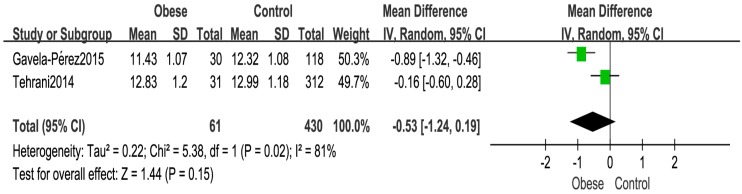
Forest plot for the age at menarche between the obese and normal weight control girls.

Meta-analysis based on 491 girls in two cohort [29,31] studies indicated that there was no statistical difference (WMD: −0.53 years, 95% CI: −1.24–0.19, 491 subjects) at the age of menarche between the obese and control groups (Figure 2). 

Another included cohort study [35], which was divided by the median of BMI (16.3 kg/m^2^), reported that eight-year-old girls with a BMI above the median had significantly earlier menarche compared with those with BMI below the median (HR: 1.65, 95% CI: 1.33–2.05) in 776 girls. The included prospective birth cohort [13] also showed that overweight/obese status at the age of 7 years was associated with increased risk of early menarche (OR = 1.79, 95% CI: 1.20–2.67).

### 3.5. Number of Girls with Menarche 

**Figure 3:**
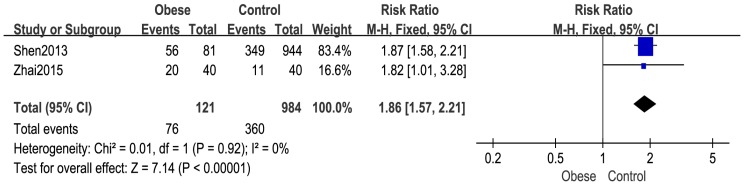
Forest plot for number of girls with menarche between the obese group and control group.

Two studies [28,30] reported the number of girls with menarche. The overall pooled estimate showed that more girls in the obese group had menarche compared with girls in the normal weight group (RR: 1.86, 95% CI: 1.57–2.21, 1105 subjects) (Figure 3).

### 3.6. Number of Girls with Early Puberty

**Figure 4:**
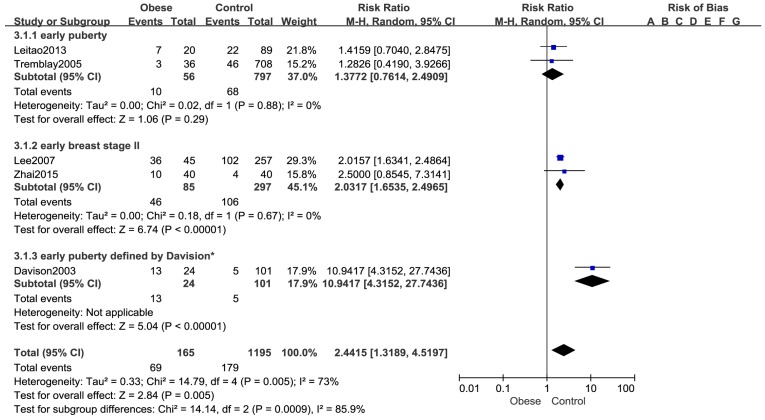
Forest plot for number of girls with early puberty between the obese group and control group.

Five cohort studies [11,12,30,32,33] reported the number of girls who had early puberty based on 1360 girls. The pooled analysis indicated that the number of girls with early puberty was significantly greater in the obese group than that in normal-weight group (RR: 2.44, 95% CI: 1.32–4.52, 1360 girls) (Figure 4). A random effects model was used (*p* = 0.005, I^2^ = 73%). Subgroup analysis showed that there was no statistical difference in the number of girls with early menarche (RR: 1.38, 95% CI: 0.76–2.49, 853 girls) between the obese and control group, while the number of girls with early breast development stage II (RR: 2.03, 95% CI: 1.65–2.50, 382 girls) in obese group was significantly higher than the number of girls in the normal weight control group, Another study by Davison [12] defined the early puberty if girls met at least two of the following three criteria: (1) highest tertile for estradiol; (2) Tanner stage 3 for breast development; and (3) highest tertile for the PDS (Pubertal Development Scale, PDS) at 9 years old. The results demonstrated that the obese group had more girls with earlier puberty than normal weight group. (RR: 10.94, 95% CI: 4.32–27.74, 125 girls). In addition, the association between obesity and early puberty was not changed in each individual sensitivity analysis by leaving one out approach. 

### 3.7. Breast Pubertal Development in Girls

One cohort study [30] comprising 80 girls reported the number of girls who had breast stage 2 development. Compared with control group, more girls in the obese group entered into breast stage 2 (RR: 1.63, 95% CI: 1.21–2.21, 80 girls).

### 3.8. Genitalia Development in Boys

One cohort study [30] included in this systematic review divided 401 boys into 1 of 3 BMI trajectories generated from the BMI z score. It reported the number of boys with genitalia development .The result of the study showed that boys in the highest BMI trajectory (mean BMI z score at age 11.5 years, 1.84) had a greater relative risk of being prepubertal (Tanner stage 1 genitalia at age 11.5) compare with boys in the lowest BMI trajectory (mean BMI z score at age 11.5 years, −0.76) (RR: 2.63, 95%CI: 1.05–6.61). 

## 4. Discussion

Puberty is regulated by the hypothalamic-pituitary-gonadal axis, which is assessed by using testicular volume and genital development in boys and breast development in girl, while adrenarche is assessed by using pubic hair development. Our systematic review and meta-analysis evaluated the relationship between obesity and puberty timing based on eleven cohort studies included the outcome measures of age of menarche, number of girls with menarche and early puberty defined by studies, breast development in girls, genitalia development in boys with a total of 4841 cases. All the outcomes included in this systematic review were related to gonadal function. Current studies showed that the potential mechanisms of obesity-promoting gonadal axis initiation mainly related to insulin resistance and hyperinsulinemia, hyperandrogens, and leptin. (1) Insulin resistance in obese subjects is associated with compensatory hyperinsulinemia and decrease levels of liver sex hormone binding protein, which increases the estrogen levels and promotes breast development [4,38,39]. (2) Obesity is often accompanied by inflammatory reactions that increase the cytokines and promote the synthesis of androgen; such changes in androgen could precipitate early pubertal development, as seen in patients with congenital adrenal hyperplasia [40]. (3) Studies showed that leptin levels are positively correlated with obesity, which may be associated with leptin resistance, while leptin also stimulates the central pulsatile gonadotropin secretion and triggers the timing of puberty by binding to receptors in the GnRH neurons [41,42,43].

The present meta-analysis did not find significant differences between two groups on age at menarche with heterogeneity of 81%, which may not necessarily mean that there is no relationship between obesity and age of menarche. The inconsistency may be related to study Tehrani [31], girls in this study who had their first period at a younger age were not considered, which leads to the menarche age older in the obese group. As another two included cohort studies [13,35] comprising 788 girls and 776 girls respectively, all showed that girls with a higher BMI at younger age had significantly earlier menarche compare with those with lower BMI, which consist with George [16] and one case-control study [44] conducted in Korea with 144 girls, demonstrating an inverse association between body fat and age at pubertal onset in girls.

As for the number of girls with menarche, the pooled estimates showed that obesity is a risk factor for early menarche, which was in agreement with Frisch [45], who proposed that the onset of the female adolescent growth spurt and menarche require a critical weight of 47.8 kg, and that increased body fat can lead to early height spurt start age and menarche age in puberty [45,46]. 

The results of subgroup analysis indicated that the association between the number of early menarche and obesity is not statistically significant which may due to lack of power, while obesity is related to early breast development of girls, which may lead to early puberty in girls. The same result was reported by another cohort [47], BMIZ (BMI-for-age z score) at 8 years were positively associated with breast development in 1135 girls. The heterogeneity of the pooled estimated decreased after conducting subgroup analysis by outcome measures, which suggest that the difference of outcome measures is one of the potential sources of heterogeneity. However, because of the limited number of studies included in each outcome measure, further cohort studies of high quality are needed to confirm the results.

Only one cohort [34] studies have evaluated the association between obesity and the timing of genitalia development in boys in our systematic review, which showed that boys with higher BMI trajectory were more likely to be later mature compared with lower BMI trajectory, which is consistent with cross-sectional studies conducted by Lee [23] and Wang [24], boys with a higher BMI were more likely to be classified as late matures. However, another cohort based on 1060 boys [47] reported that BMI-for-age z at 5 years were positively associated with pubic hair development, which consisted with the results in girls. It is hard to draw a definite conclusion that obesity led to early puberty timing in boys due to the limited number of studies with small sample size in this meta-analysis. Compared with the study of girls, there are few studies on boys, the reason may be that data can be even more difficult to interpret in boys considering that early staging of genitalia and subsequent progression through puberty (without assessment of testicular volume) is more subjective, with no easily identified event like menarche in girls [4,48]. Moreover, the focus of public attention from the beginning was mainly concentrated in the advanced puberty development in girls and ignored the boys. As there is inadequate comparable data to make conclusive decisions relating the relationship of age of pubertal onset and obesity, large longitudinal studies are needed to provide adequate comparable data to examine the relationship of puberty timing and adiposity in boys. 

To the best of our knowledge, this is the first systematic review and meta-analysis to examine the association between obesity and puberty timing in both girls and boys using only cohort studies. We conducted the present study based on a rigorous approach of a systematic review and meta-analysis. Nevertheless, several potential limitations should be acknowledged. First, several cohort studies were excluded from our meta-analysis as the outcome measures of interest were not reported [49,50] or they did not classify exposure group and non-exposed group [16,47], which may result in potential selection bias. A three years follow-up study of 8–12-year-old pupils in China [49] demonstrated that the incidence of menarche in obese children of all age groups was higher than in the normal-weight group, while the incidence of first spermatorrhea in obese children was lower than that in normal-weight group. Another cohort in Sweden [50] which involving 1901 children defined the pubertal development by childhood pubertal height growth, in which the result suggests that onset of puberty reached for girls/boys was 3.5/2.5 monthly earlier in the overweight and obese group than the normal-weight and underweight group. Second, the number of eligible cohort studies was relatively small, especially in boys, which made it difficult to draw a definite conclusion on the associations of obesity with pubertal development. Further, each included study only reported few outcome measures, heterogeneity that comes from the criteria of obesity, regional diversity, race diversity, sample size difference, age variation for each outcome measure could not be analyzed in the present analysis. Moreover, the outcome measures of included studies were not unified, which cannot be merged and analyzed.

## 5. Conclusions

In summary, our findings of the present meta-analysis suggest that obesity contributes to early onset of puberty in girls, including age at when puberty occurred. In boys, there is inadequate comparable data to make conclusive decisions the relationship of puberty timing and obesity. As there are a limited number of cohort studies included in this meta-analysis, further large-scale prospective cohorts with sufficient and reliable markers of puberty onset as well as the unified criteria for obesity are needed to validate the existence of a causal relationship, both in girls and boys.

## Figures and Tables

**Table 1:** Characteristics of studies included in the meta-analysis.

Study	Country	Sample Size Obesity/Control ^1^	Gender	Age Interval (years)	Defined Criteria for Obesity	Study Design	Primary Outcome	Quality Score
Shen 2013 [28]	China	81/944	Girls	7.02–12.02	BMI ≥ 95th percentile	Cohort	No. of girls that had menarche	8
Gavela-Pérez 2015 [29]	Spain	30/118	Girls	7.23–14.61	According to Cole [36]	Cohort	Age at menarche	8
Zhai 2015 [30]	China	40/40	Girls	8.5–12.5	Percentage of body fat using skin-fold thickness: ≥25%	Cohort	No. of girls that had menarche No. of girls with breast stage ^2^ No. of girls with early puberty ^2^	9
Ramezani Tehrani 2014 [31]	Iran	31/312	Girls	12–18	BMI > 95th percentile	Cohort	Age at menarche	8
Lee 2007 [11]	US	5/257	Girls	3–12	BMI ≥ 95th percentile	Cohort	No. of girls with early puberty ^3^	9
Leitao 2013 [32]	Portugal	20/89	Girls	7–15	≥30 % body fat	Cohort	No. of girls with early puberty ^4^	9
Tremblay 2005 [33]	Canada	36/708	Girls	11–13	BMI ≥ 95th percentile	Cohort	No. of girls with early puberty ^5^	8
Davison 2003 [12]	US	24/101	Girls	5–9	According to 2000 growth charts from the Centers for Disease Control and Prevention [37]	Cohort	No. of girls with early puberty ^6^	7

^1^ Control group: normal-weight group; ^2^ Early puberty was defined as girls who reached breast stage II earlier than the median age for that stage in China (9.2 years); ^3^ Early puberty occurred was defined as breast development at or more than breast stage II by physical examination at the grade 4visit (9.6 ± 0.1 years); ^4^ Early puberty occurred was defined according to age at menarche, <12 years for early menarche, 12–13 years for average menarche, and >13 years for late menarche; ^5^ Early puberty occurred was defined according to age at menarche, those who are below two standard deviations from their average maturing peers (1.28z to 1.04z)(z=(x-μ)/σ), or 10–15% of the left tail of the normal range distribution based on the age at which they reported having had their first menses. In this study, early menarche averaged 11 years old, on-time pubertal timing averaged 12 years old and late pubertal timing averaged 13 years; ^6^ Early puberty occurred was defined as girls who fulfilled at least 2 of the following 3 criteria: (1) highest tertile for estradiol; (2) Tanner stage 3 for breast development; and (3) highest tertile for the PDS (Pubertal Development Scale, PDS) at 9 years old.

**Table 2:** Characteristics of studies included in the systematic review but not in meta-analysis.

Study	Country	Sample Size	Gender	Age Interval (years)	The Basis of Grouping	Study Design	Outcome Measures	Results	Quality Score
Lee 2010 [34]	US	401	Boys	2–11.5	Highest BMI z score trajectories group (mean BMI z score of 1.84 (0.50) at age 11.5 years Intermediate BMI z score trajectories group (mean BMI z score of 0.41 (0.70) at age 11.5 years Lowest BMI z score trajectories group (mean BMI z score of −0.76 (0.63) at age 11.5 years	Cohort	Tanner genitalia stage	Boys in the highest BMI trajectory had a greater relative risk of being prepubertal ^1^ compare with boys in the lowest BMI trajectory (RR: 2.63, 95% CI: 1.05–6.61)	8
Deborah 2006 [35]	Australia	776	Girls	1–13	According to the median of BMI (16.3) in this study. Expose group: BMI ≥ 16.3 Control group: BMI <16.3	Cohort	Age at menarche	Eight-year-old girls with a BMI above the median had significantly earlier menarche compared with those with BMI below the median (HR: 1.65, 95% CI: 1.33–2.05)	8
Flom 2017 [13]	US	788	Girls	From birth to menarche occurred	Expose group: BMI ≥ 85th percentile Control group: BMI < 85th percentile	Cohort	Age at menarche	Overweight/obese status at the age of 7 year was associated with increased risk of early menarche ^2^ (OR = 1.79, 95% CI: 1.20–2.67)	9

^1^ Prepubertal: boys with Tanner stage 1 genitalia at age 11.5 were defined as prepubertal; ^2^ early menarche: age of menarche <12 years old.

**Table 3:** The Newcastle-Ottawa Scale (NOS) for assessing the methodology quality of cohort study.

Study	Selection				Comparability	Outcome			Score ^1^
	Representativeness of the Expose Cohort	Selection of the Non-Exposed Cohort	Ascertainment of Exposure	Demonstration That Outcome of Interest Was not Present at Start of Study	Comparability of Cohorts on the Basis of the Design or Analysis	Assessment of Outcome	Was follow-up Long Enough for Outcomes to Occur	Adequacy of Follow up of Cohorts	
Shen 2013 [28]	1	1	1	1	1	1	1	1	8
Gavela-Pérez 2015 [29]	1	1	1	1	1	1	1	1	8
Zhai 2015 [30]	1	1	1	0	2	1	1	1	8
Ramezani Tehrani 2014 [31]	1	1	1	1	1	1	1	1	8
Lee 2007 [11]	1	1	1	1	2	1	1	1	9
Leitao 2013 [32]	1	1	1	1	2	1	1	1	9
Tremblay 2005 [33]	1	1	1	0	1	1	1	1	7
Davison 2003 [12]	1	1	1	1	1	1	1	0	7
Lee 2010 [34]	1	1	1	1	2	1	1	0	8
Deborah 2006 [35]	1	1	1	1	2	1	1	0	8
Flom 2017 [13]	1	1	1	1	1	1	1	1	9

^1^ Low-quality research: scored 0–3, moderate quality research: scored 4–6, high-quality research: scored 7–9.
